# Effects of dietary rubber seed oil on production performance, egg quality and yolk fatty acid composition of Hy-Line Brown layers

**DOI:** 10.5713/ajas.19.0832

**Published:** 2020-04-12

**Authors:** Qiongfen Lu, Peifu Chen, Yan Chai, Qihua Li, Huaming Mao

**Affiliations:** 1 College of Animal Science and Technology, Yunnan Agricultural University, Kunming 650201, China; 2 Key Laboratory of Animal Nutrition and Feed Science of Yunnan Province, Kunming 650201, China; 3 College of Veterinary Medicine, Yunnan Agricultural University, Kunming 650201, China; 4 Mangshi Bureau of Science and Technology, Dehong 678400, China

**Keywords:** Average Egg Weight, Egg Production, Feed Intake, Laying Hens, Polyunsaturated Fatty Acids, Rubber Seed Oil

## Abstract

**Objective:**

This study aims to evaluate the effects of dietary supplement of rubber seed oil on production performance, egg quality, and yolk fatty acid composition in laying hens during a 16-week feeding trial period.

**Methods:**

Forty-eight 25-week-old laying hens of Hy-Line Brown were randomly divided into three groups. Each group comprised four replicates and each replicate had four birds. Rubber seed oil was incorporated into a corn-soybean meal basal diet by 3.5% (group I), 4.5% (group II), or 0 (control group) and equivalent nutrition was supplied for the test groups and the control group. The performance related values were determined using standard or well established methods.

**Results:**

No significant difference was found in the production performance, the egg quality, the composition of saturated fatty acids, and the content of cholesterol and monounsaturated fatty acids in the yolk within the three groups. Interestingly, both test groups achieved a significantly higher content of linoleic acid, α-linolenic acid, eicosapentaenoic acid, and docosahexaenoic acid and a significantly lower content of arachidonic acid (p<0.05) compared with the control group. With the increased level of dietary rubber seed oil, there was an increasing trend in the content of n-6 polyunsaturated fatty acids (PUFA), n-3 PUFA and total PUFA, but a declining trend in the n-6/n-3 ratio.

**Conclusion:**

These results demonstrate that the rubber seed oil supplemented diet effectively improved the total PUFA content in eggs without impairing the layers’ production performance and the egg quality.

## INTRODUCTION

Increasing prices of food and feed drive the exploitation of alternative non-conventional edible materials. Rubber seed is a by-product of the rubber tree (*Hevea brasiliensis*) plantations that are mainly distributed in Southeast Asia including Yunnan Highlands. According to FAO estimation, the annual yield of global rubber seed was 2.93 million tons in 2017, which makes it necessary to be rationally utilized. The content of oil in rubber seed can be as high as 450 g/kg on a dry matter basis [1]. At present, rubber seed oil is mainly used to produce biofuel. However, its nutritive value for animals and humans has been recently noticed despite the existence of hydrogen cyanide that is easily destroyed by dehydration, boiling and storage [2]. Consequently, rubber seed oil is an edible oil. Notably, the content of unsaturated fatty acids in cold-press manufactured rubber seed oil can reach up to 837 g/kg, and what’s more, it has similar physical and chemical properties to soybean oil [3]. Modification of a diet for laying hens using oilseeds other than soybean meal such as rubber seed oil and/or enzyme or antioxidant additives is expected to reduce yolk cholesterol and optimize fatty acid composition of eggs [4–6], thereby increasing polyunsaturated fatty acids (PUFA) content in eggs and providing humans with PUFA-rich eggs. In this study, two levels of rubber seed oil were added into layers’ basal diet for determination of its effects on production performance, egg quality and yolk fatty acid composition.

## MATERIALS AND METHODS

### Bird handling

The tested rubber seed oil was provided by Huakun Biotech Co., Ltd (Xishuangbanna, China). Forty-eight 21-week-old laying hens of Hy-Line Brown were purchased from a commercial layer farm located in Shilin County, Yunnan Province. The feeding experiment was conducted with the corporation’s financial support on the affiliated chicken farm and complied with the guidelines issued by IACUC of Yunnan Agricultural University. These birds were housed in a separate pen with an average indoor temperature between 20.5°C to 21.3°C and each bird was reared in a single cage. They were randomly divided into three groups and each group comprised four replicates, namely, there were four birds each replicate. The birds were fed the same diet as previous for another two weeks, the three groups were then supplied a transitional diet that comprised one of experimental diets and the former diet at the ratio of 1:2, 1:1, and 2:1 for 2 days, respectively, to reduce potential stress. Subsequently, the experimental diets were given in one-week pre-test period. Specifically, the control group was fed the basal diet, to which 3.50% or 4.50% rubber seed oil was introduced for group I and II, respectively. The formal trial began when the birds reached 25 weeks old and lasted for 16 weeks.

### Experimental diets

Each purchased raw material was the same batch. The daily diets were prepared according to the principle of each group being supplied equivalent metabolizable energy and nutrients with reference to the nutritional requirements of laying hens in the “National Chicken Feeding Standard of China” (NY/T 33-2004). The composition and the nutritional level of the experimental diets are shown in Table 1. Contents of fatty acids in the test rubber seed oil and the experimental diets were determined by high-performance liquid chromatography (HPLC) (Table 2).

### Feeding management

The pen was daily cleaned up and disinfected according to the routine procedure. The birds were fed at 7:00 and 16:00. The windows were opened before feeding in the morning to improve indoor air environment and closed before turning off the lights at night to keep a reasonable room temperature. Natural light at daytime and artificial light at night was supplied in order to satisfy 16-hour illumination each day. Free feed and water intake were guaranteed.

### Sampling

Eggs were collected at 16:30 each day on a bird individual basis and were numbered and dated in order. Average egg weight and egg production were calculated once a week. For each replicate, 10 eggs were randomly sampled weekly for measurement of egg shape index, and six eggs were further used for determination of average values of egg weight, yolk weight, eggshell weight, eggshell thickness, yolk density and eggshell density, which constituted the basic data for statistical analysis. A body weight at the end of the formal trial minus that at the start of the pre-feeding trial yielded total weight gain. When the feeding test was finished, one bird in each replicate was randomly selected and slaughtered to determine abdominal fat rate.

For each replicate, four eggs collected at the 4th and 6th weekends were merged to form a mixture sample of egg yolk after egg white was removed. The yolk samples were then stored at −20°C before determination of fatty acid composition in the yolk. At the end of week 16, four eggs were randomly selected from each group for measurement of yolk cholesterol content.

### Sample pretreatment

#### Methyl esterification of yolk fatty acid

About 5.0 grams of egg yolk was subjected to 48-hour lyophilization, 0.5000 gram yolk powder was then precisely weighed and transferred into a test tube with a stopper, followed by adding 2 mL n-hexane, then slowly dropping 3 mL of 50 mL/L freshly prepared formyl chloride. After gentle vortex for 1 min, the tube was placed in a thermostatic water bath and incubated for 2 h at 70°C. When the tube cooled to room temperature, 5 mL of 60 g/L K_2_CO_3_ solution was added, and 2 mL n-hexane was then used to make the sample approximately neutral. Shaken at a moderate speed for 30 s, the sample was centrifuged at 1,000 g for 10 min. The upper layer containing n-hexane was transferred to a new tube, into which 1 g anhydrous sodium sulfate was added for the purpose of dehydration. The extract was subjected to filtration using an organic membrane with the aperture at 0.45 μm before automatic analysis [7].

#### Extraction of yolk cholesterol

Egg yolk was homogenized in a beaker. Then 5.00 g of yolk was diluted with distilled water to a volume of 50 mL in a volumetric flask. One milliliter of the diluted yolk was then fully mixed with the same volume of 95% ethanol in a test tube, followed by sequential addition of 2.5 mL of diethyl ether and 2.5 mL of petroleum ether with at least a 30 s interval and a constant shaking. The fully shaken test tube was left to stand for about 30 min until liquid layers separated. The upper organic phase was transferred to another tube and the remaining was subjected to a second extraction. The total extract was placed in a water bath maintaining at 40°C until it was blow-dried with nitrogen, then dissolved in 2.0 mL of anhydrous ethanol and further filtrated using a nitrocellulose membrane Φ 0.45 μm before HPLC analysis [8].

### Sample analysis

#### Measurement of production performance and egg quality

According to the requirements of “Performance Terms and Measurement for Poultry” (NY/T 823-2004) issued by the Chinese Ministry of Agriculture, such parameters as egg production, feed conversion rate, daily feed intake, average egg weight, eggshell thickness, egg shape index (the ratio of length to width), eggshell weight, egg yolk weight, shell to egg ratio and yolk to egg ratio were measured during the formal trial period. Data were statistically analyzed on a weekly basis.

#### Quantification of yolk fatty acids

The quantification process referred to the method described by Sukhija and Palmquist [9] with modifications. HP-7890 gas chromatograph (Agilent, Santa Clara, CA, USA) operating conditions were as follow: 30 mm×0.25 mm×0.25 μm HP INNOWAX column, N_2_ as carrier gas; 23.371 mL/min constant column flow plus tail gas velocity, 40 mL/min H_2_ flow, 400 mL/min air flow; inlet temperature at 220°C, 2 μL splitless sampling volume, pressure at 16.205 Psi, 3 mL/min septum purge flow; flame ionization detector working temperature at 280°C. Heating procedure: hold at 100°C for 1 min, followed by 200°C for 5 min and then 250°C for 10 min. The retention time was used for qualitative analysis and the external standard method for quantitative analysis. The standard curve was plotted with concentrations of fatty acid methyl standard solution as X-axis and peak areas as Y-axis. The linear equations generated the following determination coefficients: methyl laurate (*R*^2^ = 0.999 2), methyl myristate (*R*^2^ = 0.999 4), methyl palmitate (*R*^2^ = 0.999 1), and methyl heptatanoate (*R*^2^ = 0.999 3), methyl stearate (*R*^2^ = 0.999 4), methyl oleate (*R*^2^ = 0.998 2), linoleic acid (LA) methyl ester (*R*^2^ = 0.999 0), γ-linolenic acid (GLA) methyl ester (*R*^2^ = 0.999 2), α-linolenic acid (ALA) methyl ester (*R*^2^ = 0.999 3), methyl eicosate (*R*^2^ = 0.999 8), arachidonic acid (AA) methyl ester (*R*^2^ = 0.999 2), eicosapentaenoic acid (EPA) methyl ester (*R*^2^ = 0.999 1), docosahexaenoic acid (DHA) methyl ester (*R*^2^ = 0.999 8).

#### Determination of yolk cholesterol

The HPLC method described by Yang and Dai [8] was adopted. The HP-1100 instrument (Agilent, USA) was matched with HP 20RBA× SB-C18 column (Φ 5 μm, 250 mm×4.6 mm) and the filtrated acetonitrile-isopropanol (4:1) solution was used as mobile phase at a flow speed of 1.200 mL/min, with a column temperature at 35.0°C and a detection wavelength at 210 nm. A sample volume of 10 μL was injected. Similarly, based on cholesterol standard solutions, the linear regression equation was established as follows: y = 1,977.3x+3.1296 (*R*^2^ = 0.999 8).

### Data analysis

Data were expressed as mean±standard deviation and subjected to one-way analysis of variance and Tukey’s honestly significant difference test using SPSS 17.0 software (IBM, New York, NY, USA) with a value p<0.05 indicating significant difference between groups.

## RESULTS

### The dietary rubber seed oil had no effect on the production performance of laying hens

No significant difference was found in the egg production, the feed conversion rate, the average daily feed intake, the average egg weight, the initial body weight, the final body weight and the body weight gain in the three groups (Table 3). Interestingly, both test groups had a lower abdominal fat percentage than the control group and the difference between group II and the control group was significant (p<0.05).

### The dietary supplement of rubber seed oil had no effect on the egg quality

No significant difference was detected in the eggshell thickness, the eggshell weight, the yolk weight, the shell to egg ratio, the yolk to egg ratio, or the yolk cholesterol content between any two groups (Table 4). However, the difference in egg shape index and eggshell to egg ratio between group I and the control was significant (p<0.05).

### The addition of rubber seed oil significantly influenced the yolk fatty acid composition

At week 4 and week 6, no significant difference was found in the content of lauric acid (C12:0), myristic acid (C14:0), palmitic acid (C16:0), margaric acid (C17:0), stearic acid (C18:0), and arachidic acid (C20:0) in the yolk between any two groups (Table 5), indicating that the dietary supplement of rubber seed oil did not change the composition of saturated fatty acids (SFA) in the yolk. There was also no significant difference in the content of oleic acid (C18:1) and GLA. However, a significantly higher content of LA, ALA, EPA, and DHA, and a significantly lower content of AA were detected in the test groups (p<0.05). There existed an increasing trend in the content of LA (p = 0.087), ALA (p = 0.028), EPA (p = 0.015), DHA (p = 0.036), PUFA (p = 0.079), and n-3 PUFA (p = 0.029) but not monounsaturated fatty acids in the yolk and a decreasing trend in the ratio of n-6/n-3 (p = 0.094) (data not shown).

## DISCUSSION

The attempt to utilize rubber seed oil as feed and food has occurred for decades. A joint research group working in Kunming, Yunnan, China, reported in 1981 that 6-month consumption of a diet containing 10.0% or 20.0% local rubber seed oil by mice and rats had no harmful effects on their growth, reproduction and liver function but decreased the serum lipids. Other studies showed that intake of 8.0% rubber seed oil-containing feed did not affect the growth rate, the feed consumption and the apparent digestibility of fatty acids in broilers [10] and oral administration of rubber seed oil appeared to have no obvious teratogenic and embryotoxic effects on the development of embryos in Sprague Dawley rats [11]. Thus, it is feasible to produce eggs by modifying a diet for laying hens using PUFA-rich rubber seed oil [7,12]. In this study, addition of 3.5% or 4.5% rubber seed oil to the diet did not affect the production performance of laying hens, except for a declined abdominal fat percentage. In dairy cows, supplement of 40 g/kg rubber seed oil or flaxseed oil increased the functional fatty acids such as ALA, vaccenic acid and conjugated linoleic acid and decreased SFAs in milk fat, but with phospholipids, growth hormone, insulin and beta-hydroxybutyric acid in blood unchanged [13], and even enhanced their immune function [14]. Moreover, consumption of refined rubber seed oil proved to be safe and hypolipidemic in rhesus monkeys [15] and human beings [16]. Our study further confirmed the safety of supplementing rubber seed oil to layers’ diets.

The egg quality is mainly determined by genetic, feeding and environmental factors. Egg shape index is related to egg quality characteristics and ranges from 1.30 to 1.38 [17,18]. The egg shape index obtained in this study was slightly less than 1.30, and it may be attributed to insufficient protein supply for these hens that were just in the peak period of laying eggs. The eggshell weight here accounted for 86.2 to 87.9 g/kg egg weight, which is an acceptable shell to egg ratio when compared to the normal value varying from 70 to 110 g/kg. A minor reduction in eggshell thickness will weaken the strength of eggshells and increase fragility [19]. In this study, the eggshell thickness was in the normal range (0.3 to 0.4 mm). In brief, the egg quality remains unchanged in laying hens eating the rubber seed oil supplemented diet.

It is attractive to produce table eggs containing less cholesterol and more PUFA. A recent study involving 360 birds showed that dietary supplement of rubber seed oil could effectively enrich n-3 PUFA and reduce cholesterol content in the egg yolk [6]. The process of *in vivo* cholesterol synthesis can be blocked by PUFA partially because essential fatty acids increase the solubility of cholesterol and then reduce the amount of cholesterol deposited in the yolk [20]. However, the reduction of yolk cholesterol content in the test groups fed dietary rubber seed oil was statistically insignificant presumably because of the smaller bird and yolk sample sizes analyzed in this study. In spite of this, we found that the inclusion of 35.0 or 45.0 gram rubber seed oil per kg in the basal diet for laying hens significantly increased the content of PUFA such as LA, ALA, EPA, and DHA and significantly decreased the n-6/n-3 ratio in egg yolk. Increasing the PUFA content in chicken diets by adding fish oil, flaxseed product or microalgae promoted the deposition of PUFA in the egg yolk [21–26], which is consistent to the results of this study and Wen’s study using rubber seed oil [6]. It may be due to the competitive inhibition of AA synthesis or deposition in eggs by n-3 PUFA through mechanisms including preferential binding to Δ5 desaturase [27–29]. Therefore, the results of this study and the other studies confirmed that supplement of rubber seed oil increased egg yolk PUFA. Furthermore, considering the health benefit of n-3 PUFA consumption on inflammation and metabolic syndrome prevention and treatment in humans [30], the decreased n-6/n-3 ratio in egg yolk in the test groups compared to the control group indicated that dietary supplement of rubber seed oil would be of practical value in layer farming.

In conclusion, supplementing 3.5% or 4.5% rubber seed oil to the corn-soybean meal basal diet significantly increased PUFA content and reduced n-6/n-3 ratio in egg yolk without undesirable effects on the production performance and the egg quality.

## Figures and Tables

**Table 1:** The composition and the nutrient level of the experimental diets (air dry matter %)

Items	Groups1)		

	Control	I	II
Ingredients			
Corn	56.57	54.05	49.34
Soybean meal	26.92	26.21	25.47
Wheat bran	1.46	4.72	9.24
Lard	3.50	0	0
Rubber seed oil	0	3.50	4.50
Sodium chloride	0.35	0.35	0.35
Limestone	8.49	8.56	8.64
Calcium monophosphate	1.61	1.49	1.34
Lysine	0	0.01	0.01
Methionine	0.10	0.11	0.11
Premix2)	1.00	1.00	1.00
Total	100.00	100.00	100.00
Nutrient levels3)			
Metabolizable energy (MJ/kg)	11.71	11.71	11.71
Crude protein	16.50 (16.71)	16.50 (16.53)	16.50 (16.60)
Calcium	3.50 (3.88)	3.50 (3.79)	3.50 (3.68)
Total phosphorus	0.60 (0.59)	0.60 (0.62)	0.60 (0.60)
Available phosphorus	0.38	0.36	0.34
Sodium chloride	0.37	0.37	0.37
Lysine	0.83	0.83	0.83
Methionine	0.36	0.36	0.36
Ether extract	(6.60)	(7.80)	(7.40)
Crude fiber	(3.50)	(4.20)	(3.90)

1) The experimental diets introduced by 0, 3.5% or 4.5% rubber seed oil were supplied for birds in the control group, group I and group II, respectively. The nutrition level of the diets remained equivalent.

2) Premix provides the following per kg of diets: Vit A 16,500 IU, Vit D_3_ 3,300 IU, Vit E 200 mg, Vit K_3_ 3 mg, Vit B_1_ 1.5 mg, Vit B_2_ 6.0 mg, calcium pantothenate 9.0 mg, niacin 18.0 mg, Vit B_6_ 3.0 mg, Vit B_12_ 0.009 mg, folic acid 0.6 mg, biotin 0.03 mg, Zn 35.00 mg, Cu 4.00 mg, Fe 60.00 mg, Mn 30.00 mg, I 0.35 mg, Se 0.10 mg, ethoxyquin 750 mg.

3) Measured values are given in the brackets, while the others are calculated values.

**Table 2:** The fatty acid composition of the rubber seed oil and the experimental diets (mg/g)

Ingredients	Rubber seed oil	Groups1)		

		Control	I	II
Lauric acid (C12:0)	0.13	0.06	0.05	0.09
Myristic acid (C14:0)	0.02	0.26	0.23	0.31
Palmitic acid (C16:0)	0.14	0.13	0.16	0.12
Margaric acid (C17:0)	9.54	2.03	2.41	3.54
Stearic acid (C18:0)	0.34	0.11	0.13	0.17
Oleic acid (C18:1)	21.89	9.52	8.93	9.68
Linoleic acid (C18:2, n-6)	119.56	23.04	29.01	36.56
γ-Linolenic acid (C18:3γ, n-6 )	0.56	ND	ND	ND
α-Linolenic acid (C18:3α, n-3)	28.87	6.92	10.26	12.97
Arachidic acid (C20:0)	0.09	0.14	0.16	0.21
Arachidonic acid (C20:4, n-6)	ND	ND	ND	ND
Eicosapentaenoic acid (C20:5, n-3)	0.28	ND	ND	ND
Docosahexaenoic acid (C22:6, n-3)	0.49	ND	ND	ND

ND, not detected.

1) The experimental diets introduced by 0, 3.5%, or 4.5% rubber seed oil were supplied for birds in the control group, group I and group II, respectively. Measured values of fatty acids in the rubber seed oil and the diets were listed.

**Table 3:** The production performance of laying hens ingesting diets with or without rubber seed oil

Parameters	Bird sample size (n)	Groups1)			SEM	p-value

		Control	I	II		
Egg production (egg/hen/d)	16	0.970	0.969	0.965	0.003	0.73
Average feed conversion rate (kg/kg)	16	1.83	1.84	1.91	0.02	0.34
Average daily feed intake (g/hen)	16	105.81	105.95	106.02	0.60	0.99
Average egg weight (g)	16	60.38	60.34	60.46	0.25	0.98
Initial body weight (kg)	16	1.59	1.60	1.60	0.02	0.95
Final body weight (kg)	16	1.84	1.83	1.84	0.02	0.92
Body weight gain (g)	16	246.74	222.82	245.17	15.96	0.80
Abdominal fat (g/kg)	4	60.6^a^	48.1^ab^	29.9^b^	4.86	0.01

SEM, standard error of the mean.

1) The experimental diets introduced by 0, 3.5%, or 4.5% rubber seed oil were supplied for birds in the control group, group I and group II, respectively.

ab In the same row, values with no letter or the same letter superscripts mean no significant difference, while values with different lowercase letter superscripts mean significant difference (p<0.05).

**Table 4:** Effect of the dietary rubber seed oil on the egg quality on a whole trial period basis

Parameters	Egg sample size	Groups1)			SEM	p-value

		Control	I	II		
Eggshell thickness (mm)	384	0.338	0.337	0.342	0.002	0.64
Egg shape index	640	1.27^b^	1.29^a^	1.28^ab^	0.003	0.01
Eggshell weight (g)	384	5.31	5.22	5.27	0.030	0.52
Yolk weight (g)	384	14.00	14.05	14.07	0.119	0.97
Eggshell to egg ratio (g/kg)	384	87.9^a^	86.2^b^	87.1^ab^	0.027	0.04
Yolk to egg ratio (g/kg)	384	231.8	232.4	231.3	0.102	0.91
Average cholesterol content in yolk (mg/g)	4	11.30	11.02	9.92	0.365	0.29
Average cholesterol content per egg (mg/egg)	4	182.98	173.45	156.69	8.35	0.47

SEM, standard error of the mean.

1) The experimental diets introduced by 0, 3.5%, or 4.5% rubber seed oil were supplied for birds in the control group, group I and group II, respectively.

ab In the same row, values with no letter or the same letter superscripts mean no significant difference, while values with different lowercase letter superscripts mean significant difference (p<0.05).

**Table 5:** Effect of the dietary rubber seed oil levels on the content of fatty acids deposited in yolk

Fatty acids	Week	Groups1) (mg/g)			SEM	p-value

		Control	I	II		
Lauric acid (C12:0)	4	0.22	0.23	0.19	0.01	0.33
	6	0.14	0.15	0.17	0.01	0.34
Myristic acid (C14:0)	4	0.01	0.01	0.01	0.01	0.86
	6	0.01	0.01	0.01	0.00	0.63
Palmitic acid (C16:0)	4	0.60^a^	0.51^b^	0.50^b^	0.02	0.04
	6	0.65	0.53	0.53	0.03	0.23
Margaric acid (C17:0)	4	27.44	26.13	27.31	0.54	0.67
	6	31.67	31.64	32.88	1.28	0.94
Stearic acid (C18:0)	4	1.03	1.05	1.28	0.06	0.14
	6	1.43	1.15	1.31	0.06	0.18
Oleic acid (C18:1)	4	71.29	70.94	72.91	0.89	0.73
	6	88.01	88.71	90.11	1.64	0.92
Linoleic acid (C18:2, n-6)	4	18.01^c^	24.29^b^	32.38^a^	2.67	0.01
	6	23.07^c^	34.37^b^	40.57^a^	3.27	0.01
γ-Linolenic acid (C18:3γ, n-6 )	4	0.17	0.18	0.17	0.01	0.33
	6	0.20	0.191	0.21	0.02	0.50
α-Linolenic acid (C18:3α, n-3)	4	0.40^c^	3.25^b^	5.77^a^	0.98	<0.01
	6	0.52^b^	4.85^a^	6.40^a^	1.15	0.01
Arachidic acid (C20:0)	4	0.29	0.23	0.24	0.01	0.36
	6	0.34	0.26	0.25	0.02	0.17
Arachidonic acid (C20:4, n-6)	4	3.42^a^	2.79^b^	2.78^b^	0.14	0.05
	6	4.17^a^	3.11^b^	3.28^b^	0.23	0.05
Eicosapentaenoic acid (C20:5, n-3)	4	0.11^b^	0.17^a^	0.21^a^	0.02	0.01
	6	0.13^b^	0.21^a^	0.23^a^	0.02	0.02
Docosahexaenoic acid (C22:6, n-3)	4	1.35^b^	3.47^a^	4.54^a^	0.60	0.01
	6	1.59^b^	3.94^a^	4.83^a^	0.62	0.01
Saturated fatty acids	4	29.59	28.16	29.53	0.60	0.66
	6	34.24	33.74	35.15	1.33	0.94
Monounsaturated fatty acids	4	71.29	70.94	72.91	0.88	0.73
	6	88.01	88.71	90.11	1.64	0.92
Polyunsaturated fatty acids	4	23.46^c^	34.15^b^	45.85^a^	4.15	0.01
	6	29.68^c^	46.63^b^	55.25^a^	4.84	<0.01
n-6 Polyunsaturated fatty acids	4	21.60^c^	27.26^b^	35.33^a^	2.57	0.01
	6	27.44^c^	37.63^b^	43.82^a^	3.09	<0.01
n-3 Polyunsaturated fatty acids	4	1.86^c^	6.89^b^	10.52^a^	1.60	<0.01
	6	2.24^b^	9.00^a^	11.43^a^	1.78	0.01
n-6/n-3	4	11.61^a^	3.96^b^	3.36^c^	1.69	<0.01
	6	12.25^a^	4.18^b^	3.83^b^	1.74	<0.01

SEM, standard error of the mean.

1) The experimental diets introduced by 0, 3.5%, or 4.5% rubber seed oil were supplied for birds in the control group, group I and group II, respectively.

a–c In the same row, values with no letter or the same letter superscripts mean no significant difference, while values with different lowercase letter superscripts mean significant difference (p<0.05).
